# PEG-b-PCL Micelles as Nanocarriers for Poorly Soluble Benzimidazoles: A Comparative Study of Albendazole and Fenbendazole

**DOI:** 10.3390/molecules31122070

**Published:** 2026-06-12

**Authors:** Rayna Bryaskova, Gergana Krumova, Kameliya Anichina, Damyan Ganchev, Teodor Todorov, Rumiana Tzoneva

**Affiliations:** 1 Department of Polymer Engineering, University of Chemical Technology and Metallurgy, 8 Kliment Ohridsky Str., 1756 Sofia, Bulgaria; gergana7k@abv.bg; 2 Department of Organic Synthesis, University of Chemical Technology and Metallurgy, 8 Kliment Ohridsky Str., 1756 Sofia, Bulgaria; 3 Department of Machine Elements and Non-Metal Constructions, Technical University of Sofia, 1000 Sofia, Bulgaria; ganchev_d@tu-sofia.bg; 4 Institute of Biophysics and Biomedical Engineering, Bulgarian Academy of Sciences, Acad. G. Bonchev Str., Bl. 21, 1113 Sofia, Bulgaria; teo1yonkov1todorov@gmail.com (T.T.); tzoneva@bio21.bas.bg (R.T.)

**Keywords:** PEG-b-PCL micelles, albendazole, fenbendazole, drug delivery system, anticancer activity, MDA-MB-231 cells

## Abstract

Poly(ethylene glycol)-block-poly(ε-caprolactone) (PEG-b-PCL) copolymer micelles have emerged as promising drug delivery systems for enhancing the solubility and bioavailability of poorly water-soluble benzimidazole drugs. In this study, we prepared and characterized PEG-b-PCL micelles to encapsulate poorly water-soluble anthelmintics such as albendazole (ABZ) and fenbendazole (FBZ), with a focus on comparing their encapsulation behaviour, release profiles, and biological activity in cancer therapy. Drug-loaded micelles were analysed using dynamic light scattering (DLS), which revealed uniform nanosized micelles with a narrow polydispersity index (PDI). The morphology and size of both empty and drug-loaded micelles were examined using transmission electron microscopy (TEM), confirming that the micelles were spherical and consistent in size. Both drugs were efficiently encapsulated within the micellar core, demonstrating a high loading capacity. The release profiles of PEG-b-PCL micelles containing albendazole (ABZ) and fenbendazole (FBZ) at pH 7.4 were also evaluated. FBZ exhibited slower release kinetics compared to ABZ, likely due to its higher lipophilicity and stronger interactions with the hydrophobic PCL core, resulting in enhanced retention within the micelles. In contrast, ABZ had faster release kinetics. Finally, the in vitro MTT assays performed on the highly invasive triple-negative breast cancer (TNBC) cell line revealed the potential of these micelles as effective drug delivery systems.

## 1. Introduction

Cancer is one of the greatest threats to public health, causing millions of deaths annually, with the number of new cases increasing each year. Breast cancer (BC) continues to be the second leading cause of cancer-related mortality, with nearly 90% of these deaths linked to metastatic breast cancer (mBC) [1]. Microtubule-targeting agents (MTAs) are still the primary standard for treating mBC [2]. The most commonly used MTAs are paclitaxel, which stabilizes microtubules, and vincristine, which destabilizes them, both used in advanced-stage BC treatment [2,3,4]. Chemoresistance and side effects limit current MTAs [2,3], and failure of initial treatment often worsens responses to subsequent MTAs, with response rates from 12% to 35% [5]. New, less toxic MTAs with different MOAs are urgently needed. Benzimidazoles such as albendazole (ABZ), mebendazole (MBZ), fenbendazole (FBZ), and oxfenbendazole (OBZ) are a versatile class of synthetic organic compounds, originally developed as anthelmintics, that have demonstrated potent anticancer effects in preclinical models [6]. Benzimidazoles closely resemble naturally occurring purine nucleotides (the building blocks of DNA and RNA) [7]. This structural similarity allows them to cross cell membranes easily and interact with key biological targets without triggering immediate efflux from the cell. They work primarily by binding to cellular proteins, most notably beta-tubulin [8]. They accumulate within cells, disrupting essential biological processes such as mitosis, cell division, and DNA synthesis, thereby triggering programmed cell death (apoptosis) [9,10] and inhibiting tumour growth and angiogenesis via suppression of VEGF and HIF-1α expression [11,12,13].

However, ABZ and its derivatives are classified as BCS Class II drugs, characterized by high permeability but extremely low solubility in water, which leads to low and variable oral bioavailability [14,15]. To address these limitations, various formulation strategies, such as nanocrystals [16], liposomes [17], cyclodextrins [18], inclusion complexes [19], or synthetic salts [14], have been investigated. In recent years, polymeric micelles have emerged as promising drug delivery systems, attracting increasing attention as a means of targeted therapy in cancer treatment [20,21,22]. These nanosystems, composed of a hydrophobic core and a hydrophilic shell, are a key factor in the solubilization of poorly water-soluble drugs, which enhances their bioavailability and therapeutic efficacy. The hydrophobic core of the micelles serves as a site for loading highly hydrophobic drug molecules, and the polymer shell ensures the steric stabilization of the micelles, thereby protecting them from degradation and facilitating their transport through biological barriers. The most explored strategy relies on passive drug targeting via the enhanced permeability and retention (EPR) effect, which allows these nanosized micelles to accumulate effectively in solid tumours due to leaky vasculature and poor lymphatic drainage [20].

Presently, there is limited literature available on the preparation of polymer micelles loaded with benzimidazole drugs. Recently, FBZ was successfully encapsulated into polymeric micelles based on polyvinyl caprolactam–polyvinyl acetate–polyethylene glycol graft copolymer (PCL-PVAc-PEG) (Soluplus^®^). Cytotoxicity and clonogenic assays were conducted using A549 human non-small cell lung cancer cells treated with both free FBZ and FBZ-loaded Soluplus^®^ micelles. The results showed that the Soluplus^®^ micelles encapsulating fenbendazole exhibited good anticancer efficacy and have potential applications in cancer therapy [23]. In another system, ABZ and paclitaxel (PTX) were encapsulated in polymer-mixed micelles based on D-tocopheryl polyethylene glycol 1000 succinate–folic acid complex (TPGS-Fol) and Soluplus, and their cytotoxicity activity against SKOV3 ovarian cell lines was then tested [24]. Active targeting micelle systems based on RGD-decorated poly[poly(ethylene glycol) methyl ether methacrylate]-block-poly(methyl methacrylate) micelles for encapsulation of ABZ were also developed, and their potential for the treatment of ovarian cancer was demonstrated [25].

In this study, we developed polyethylene glycol-b-poly(ε-caprolactone) (PEG-b-PCL) polymeric micelles encapsulating ABZ and FBZ and evaluated their potential against the highly aggressive MDA-MB-231 triple-negative breast cancer cell line. Although structurally related, ABZ and FBZ differ in their molecular weight, lipophilicity, and hydrophobic interactions, which may influence their encapsulation behaviour, micellar retention, release kinetics, and biological activity.

PEG-b-PCL block copolymers (FDA-approved) are among the most extensively investigated polymeric nanocarriers for passive drug delivery owing to their excellent biocompatibility, controlled biodegradability, and intrinsic ability to self-assemble into nanoscale micelles in aqueous environments [26]. Their core–shell architecture is particularly advantageous: the hydrophobic PCL core enables high loading of poorly soluble anticancer agents, while the PEG corona provides steric stabilization and prolonged circulation [27]. Importantly, PEG-containing amphiphilic block copolymers significantly enhance the stability of these nanoparticles in systemic circulation and promote their selective accumulation in tumour tissues. Moreover, PEG’s ability to avoid recognition by the reticuloendothelial system improves drug availability at the target site. Consequently, this mechanism not only maximizes the therapeutic potential of anticancer drugs but also enhances the likelihood of effectively reaching and treating the targeted tumours [28].

Despite these advantages, the application of PEG-b-PCL block copolymer micelles for the encapsulation of benzimidazole carbamate drugs remains underexplored. Therefore, the aim of the present study was not only to develop PEG-b-PCL micelles for the delivery of poorly water-soluble benzimidazole derivatives but also to comparatively investigate how differences in the physicochemical characteristics of ABZ and FBZ affect micellar encapsulation efficiency, particle properties, drug release behaviour, and in vitro anticancer activity.

## 2. Results and Discussion

### 2.1. Preparation and Characterization of Nanosized Micelles with Embedded ABZ or FBZ

ABZ and FBZ drugs are known for their poor water solubility, ranging from 0.2 to 1.3 µg/mL for ABZ and 0.3 µg/mL for FBZ, which significantly limits their clinical application. Their poor solubility results in low, highly variable oral bioavailability (<5%), primarily due to dissolution-limited drug absorption [29]. One effective approach to improve their water solubility is to encapsulate them in polymer micelles. The strategy relies on the use of PEG-b-PCL copolymer as a polymer nanocarrier, which consists of a hydrophilic PEG block forming the micelle shell and a hydrophobic PCL block forming the micelle core.

**Figure 1:**
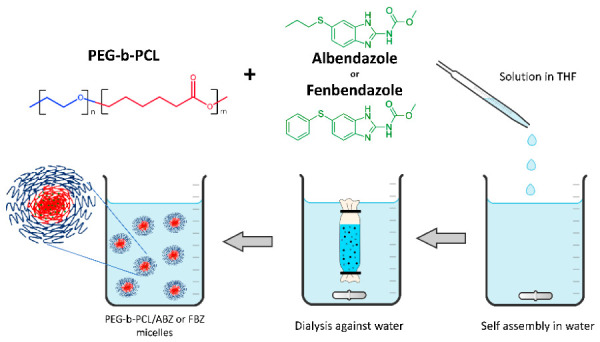
Strategy for preparation of ABZ- or FBZ-loaded micelles.

Drug-loaded micelles were prepared by dissolving the PEG-b-PCL block copolymer and the drug in a solvent that dissolves both components, such as THF (Figure 1). The solution was then added to an aqueous medium, yielding well-defined micelles. These micelles consist of a PCL core with ABZ or FBZ physically entrapped via hydrophobic interactions between the PCL block and the drug and a PEG shell that ensures polymer stabilization. High encapsulation efficiencies (EE) of ABZ and FBZ within PEG-b-PCL micelles were 96.8% (3.65 × 10^−4^ mol/L) and 98% (3.27 × 10^−4^ mol/L), respectively, as determined using Equation (1).

**Figure 2:**
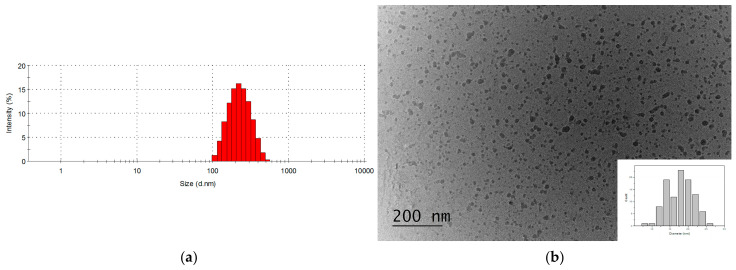
(**a**) DLS size distribution and (**b**) TEM image of PEG-b-PCL micelles.

The size and shape of non-loaded and loaded PEG-b-PCL polymer micelles were determined by dynamic light scattering (DLS) and transmission electron microscopy (TEM). The DLS analysis (Figure 2a) shows that the average hydrodynamic diameter (Dh) of non-loaded PEG-b-PCL micelles is 165 nm, with a polydispersity index (PDI) of 0.3.

**Figure 3:**
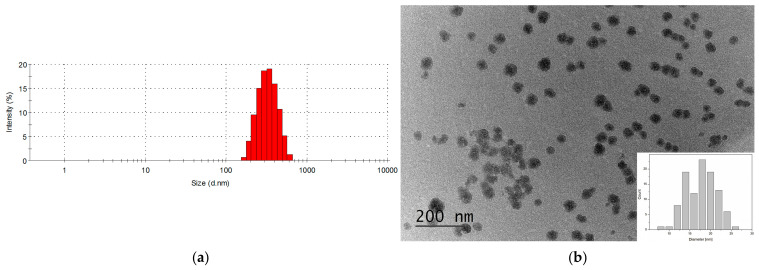
(**a**) DLS and (**b**) TEM analysis of PEG-b-PCL/ABZ micelles.

**Figure 4:**
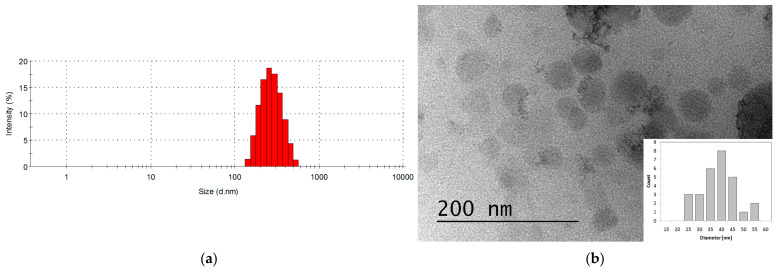
(**a**) DLS and (**b**) TEM analysis of PEG-b-PCL/FBZ micelles.

In comparison, DLS analysis of the ABZ- or FBZ-loaded PEG-b-PCL micelles (Figure 3a) reveals an increase in the hydrodynamic diameter, with a Dh of 194 nm at a polydispersity index of 0.19 for PEG-b-PCL/ABZ micelles and a Dh of 268 nm at a polydispersity index of 0.153 for PEG-b-PCL/FBZ micelles (Figure 4a).

The increased hydrodynamic diameter of the drug-loaded micelles is a result of encapsulation of hydrophobic ABZ or FBZ drugs into the hydrophobic core of the micelles, facilitated by hydrophobic interactions between the drug molecules and the PCL block, which is further confirmed by the TEM analysis.

The performed TEM analysis demonstrated the formation of spherical ABZ- or FBZ-loaded PEG-b-PCL micelles with a more contrasted hydrophobic core (Figure 3b and Figure 4b) due to the entrapped hydrophobic drugs. This observation is consistent with the low aqueous solubility of the drugs used and their affinity toward hydrophobic environments, in contrast to the non-loaded PEG-b-PCL micelles (Figure 2b). The frequency histogram of the size of the micelles obtained by TEM analysis revealed that the average diameter of the unloaded micelles is 25 nm (Figure 2b). Average diameters of 35 nm for ABZ-loaded micelles (Figure 3b) and 40 nm for FBZ-loaded micelles (Figure 4b) were measured by TEM analysis. Importantly, the observed spherical morphology differs from the irregular or angular structures typically associated with crystalline drug particles, supporting the formation of drug-loaded polymeric micellar nanostructures. The difference in the diameters observed by DLS and TEM for unloaded and drug-loaded micelles is likely due to the different physical states analysed by the two techniques. DLS measures the hydrodynamic diameter of hydrated micelles that are dispersed in an aqueous medium, including the solvated polyethylene glycol (PEG) corona. In contrast, TEM analysis is conducted in the dry state, where some dehydration and collapse of the PEG shell may occur during sample preparation. This can result in smaller apparent particle sizes.

**Figure 5:**
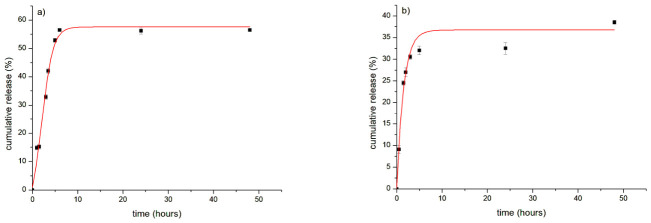
(**a**) Drug release profile of PEG-b-PCL/ABZ micelles at pH 7.4; (**b**) drug release profile of PEG-b-PCL/FBZ micelles.

The drug release test was performed at pH 7.4 and 37 °C to mimic physiological conditions. The concentration of released ABZ or FBZ in the medium was determined spectrophotometrically by measuring absorbance at 290 nm. In both cases, after an initial burst release of the drug, which was more pronounced for ABZ compared to FBZ, a phase of significantly sustained drug release over a long period (48 h) was observed (Figure 5).

The initial burst release is commonly observed in polymeric micellar systems, where part of the drug may be located near the core–corona interface or within the corona region. Drug molecules localized in these regions can diffuse rapidly into the surrounding medium without passing through the entire hydrophobic core [30,31]. In some cases, burst release may be beneficial, providing an initial therapeutic dose followed by prolonged release [29,31]. Control experiments performed with free ABZ and FBZ under identical release conditions showed rapid precipitation of the non-encapsulated drugs in the aqueous buffer medium, confirming their extremely low water solubility. In contrast, PEG-b-PCL micelles maintained colloidal stabilization of both drugs and enabled sustained release over prolonged incubation times. In addition, no visible drug crystallization or precipitation was observed in the drug-loaded micellar dispersions during the experiments.

The overall cumulative release over 48 h for PEG-b-PCL/FBZ micelles was approximately 35–40% at pH 7.4, whereas the overall cumulative release for PEG-b-PCL/ABZ micelles was slightly higher, and it was approximately 55–57%. This difference is attributed to the higher lipophilicity of FBZ (logP ≈ 3.9–4.0) compared to ABZ (logP ≈ 3.0), which promotes stronger interactions with the hydrophobic PCL core, resulting in enhanced retention within the micelles and slower diffusion into the surrounding medium.

These findings suggest that differences in drug lipophilicity significantly influence micellar retention and release kinetics, which may subsequently affect intracellular drug availability and the observed cytotoxic response.

### 2.2. Effect of ABZ- and FBZ-Loaded PEG-b-PCL Micelles on the Behaviour of MDA-MB-231 Cells

**Figure 6:**
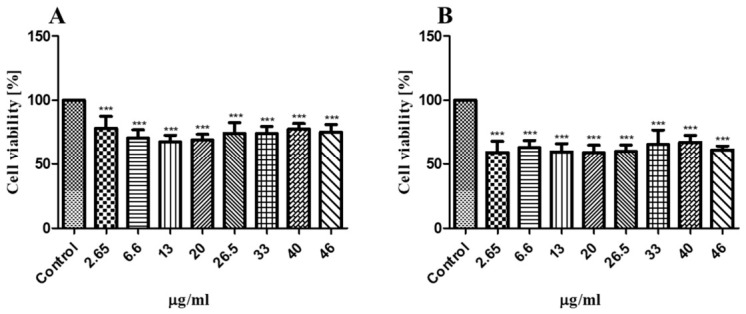
Cell viability of MDA-MB-231 cells treated with free ABZ after 24 h (**A**) and 48 h (**B**) of incubation. Data are presented as mean ± SD from three independent experiments. ***—*p* < 0.0001 (highly significant) (one-way ANOVA, Dunnett’s multiple-comparison test).

**Figure 7:**
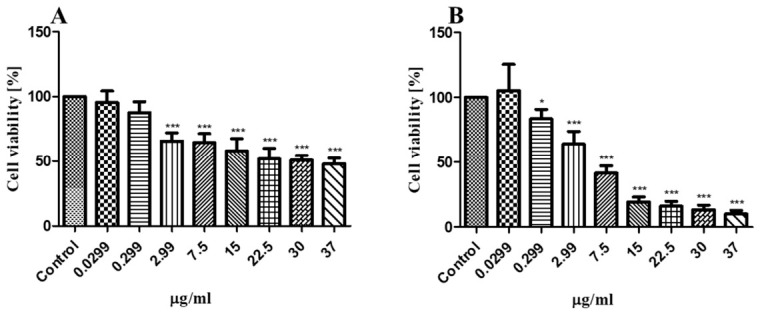
Cell viability of MDA-MB-231 cells treated with free FBZ after 24 h (**A**) and 48 h (**B**) of incubation. Data are presented as mean ± SD from three independent experiments. * (statistical significance)—***—*p* < 0.0001 (highly significant) (one-way ANOVA, Dunnett’s multiple-comparison test).

**Figure 8:**
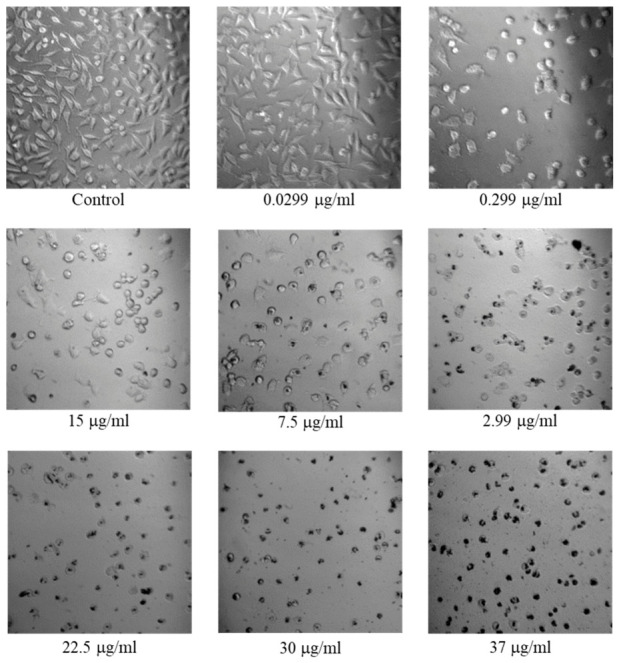
Phase contrast images of MDA-MB231 exposed to FBZ in different concentrations for 24 h. Images were taken at 20× magnification.

**Figure 9:**
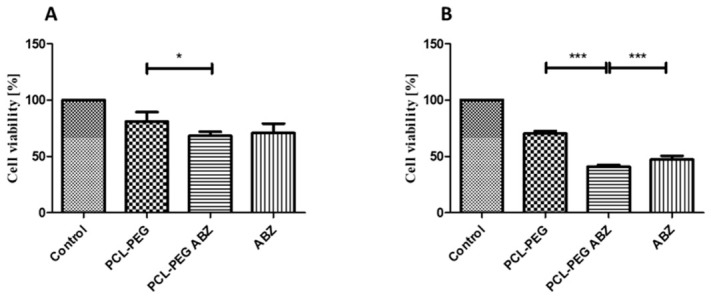
Cell viability of MDA-MB-231 cells treated with free ABZ, PEG-b-PCL micelles, and PEG-b-PCL micelles loaded with ABZ after 24 h (**A**) and 48 h (**B**) of incubation. Data are presented as mean ± SD from three independent experiments. * (statistical significance)—***—*p* < 0.0001 (highly significant) (one-way ANOVA, Dunnett’s multiple-comparison test).

The cytotoxicity of drugs, micelles, and drug-loaded micelles was evaluated using the MTT assay in the breast cancer cell line MDA-MB-231 (Figure 6, Figure 7, Figure 8 and Figure 9).

The toxic effects of ABZ and FBZ at different concentrations on the breast cancer cell line MDA-MB-231 were examined after 24 h and 48 h (Figure 6 and Figure 7). When ABZ was applied at concentrations of 2.65 μg/mL–46 μg/mL, a steady decline in cell viability was observed, ranging from 22% to 33% at 24 h (Figure 6A) and from 33% to 41% at 48 h (Figure 6B). Our results are in agreement with those of Javdan et al. [29], who showed significant toxicity in the range of 27% in MDA-MB-231, mostly observed above 10 µM (2.65 µg/mL) of ABZ. Prolonged ABZ treatment for 48 h slightly increased cancer cell sensitivity to the drug (decreasing viability by 33–41%) within the tested concentration range but again showed no concentration-dependent trend. The triple-negative breast cancer cells MDA-MB-231 were found to be much more resistant to the toxic effects of ABZ than other cancer lines, such as MCF-7, B16F10, and different prostate cancer cell lines, in which significantly higher toxicity of ABZ was achieved at much lower doses of the drug [29,32].

In contrast, treatment of the triple-negative breast cancer cell line MDA-MB-231 with FBZ showed a concentration-dependent increase in cellular toxicity (Figure 7), which allowed the calculation of IC_50_ values for 24 h and 48 h of treatment. The IC_50_ for 24 h of incubation was 29.05 µg/mL, and with an increased incubation time (48 h), the IC_50_ dropped to 7.439 µg/mL. The dose-dependent toxicity of FBZ was shown by Wang et al. in human breast, colon, and cervical cancer cells [33]. The gradual decrease in cell viability after treatment with FBZ, together with the higher cell toxicity observed in the results, in comparison with the steady decline in cell viability after treatment with ABZ, is most likely due to the higher hydrophobicity of the FBZ molecule (the higher lipophilicity of FBZ (logP ≈ 3.9–4.0) compared to ABZ (logP ≈ 3.0) [33], which could increase the retention time in the cell membrane during passive transcellular uptake [34] and thus improve the cytotoxic capacity.

The morphology of the treated cells (Figure 8) confirmed a gradual reduction in cell viability following FBZ treatment. As drug concentration increased, a decrease in cell number was observed, along with significant cell rounding, shrinkage, and black staining, most likely from the drug conglomerates.

To increase cellular uptake of poorly soluble ABZ and FBZ, they were encapsulated in PEG-b-PCL micelles. The final concentration of the drugs encapsulated in micelles and loaded into the culture medium for treatment was 10 µg/mL, as described in the Section 3. As shown above (Figure 6 and Figure 7), this concentration was sufficient to induce cytotoxicity. Generally, the cytotoxic effect of ABZ-loaded micelles increased with longer incubation periods (Figure 9A,B). Initially, after 24 h, the viability of cells treated with ABZ-loaded micelles decreased to approximately 60%. Additionally, cell viability with ABZ treatment alone did not differ from that of ABZ loaded into micelles. After 48 h, viability in cells treated with ABZ-loaded micelles decreased further to around 40%, which was statistically lower than that of ABZ alone. The higher toxicity of ABZ loaded in micelles compared with free ABZ can be explained by increased permeability of the drug-loaded micelles across the cell membrane and cumulative drug release into the cytoplasm.

**Figure 10:**
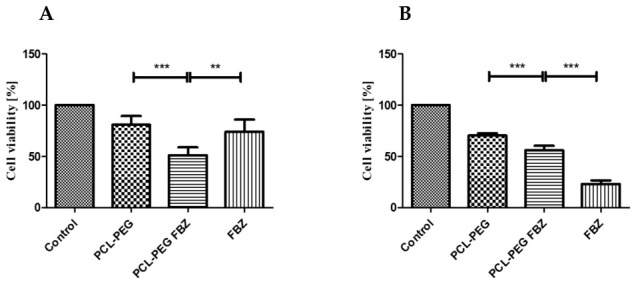
Cell viability of MDA-MB-231 cells treated with free FBZ, PEG-b-PCL micelles, and PEG-b-PCL micelles loaded with FBZ after 24 h (**A**) and 48 h (**B**) of incubation. Data are presented as mean ± SD from three independent experiments. **—*p* < 0.05 (statistically significant) and ***—*p* < 0.0001 (highly significant) (one-way ANOVA, Dunnett’s multiple-comparison test).

In contrast, the cytotoxic effect of FBZ-loaded micelles was more pronounced at the shorter incubation time (Figure 10A, B). After 24 h, the viability of cells treated with drug-loaded micelles decreased to approximately 50%, whereas the cell viability with FBZ-alone treatment remained higher at around 80%. After 48 h, the viability of cells treated with drug-loaded micelles remained nearly unchanged, whereas the viability of FBZ-treated cells dropped sharply to approximately 25%. Most likely, the lower drug release from the micelle (Figure 5b) and its reduced accumulation within the cell lead to decreased toxicity during long-term exposure.

Research indicates that benzimidazoles (including ABZ and FBZ) attach to a unique site on mammalian tubulin (the colchicine-binding site (COL site)) distinct from locations targeted by other microtubule inhibitors such as vinca alkaloids or paclitaxel [35,36,37]. As a result of this mechanism, agents like ABZ continue to exert antiproliferative effects in cancer cells that are resistant to drugs like paclitaxel [38]. The efficacy of benzimidazoles in disrupting microtubule organization was also shown in our previous study [39]. We observed that benzimidazole-2-yl hydrazone derivative-loaded micelles significantly enhanced antiproliferative and cytotoxic effects on MDA-MB-231 cells, with sustained impacts on microtubule disruption, apoptotic changes, and localization in the perinuclear area, similar to nocodazole (synthetic benzimidazole-based antimitotic agent), which interferes with tubulin dynamics by binding to the beta-tubulin subunit [40].

### 2.3. Kinetic Modelling of In Vitro Drug Release

To further elucidate the release mechanism of ABZ and FBZ from PEG-b-PCL micelles, the experimental cumulative release data were analysed using the Higuchi and Korsmeyer–Peppas models applied to the initial burst phase (≤60% release).

The Higuchi model (Equation (1)) describes diffusion-controlled release:

Q = k_H_t^1/2^ (1)

where Q is the cumulative amount of drug released, and k_H_ is the Higuchi dissolution constant.

The Korsmeyer–Peppas model (Equation (2)) was used to determine the underlying release mechanism:

M_t_/M_∞_ = kt^n^ (2)

where M_t_/M_∞_ is the fraction of drug released at time t, k is the kinetic constant, and n is the release exponent indicative of the transport mechanism. For spherical geometry, n ≤ 0.43 indicates Fickian diffusion, 0.43 < n < 0.85 corresponds to anomalous (non-Fickian) transport, and n ≥ 0.85 suggests Case-II relaxation/erosion.

Kinetic parameters are summarized in Table 1.

ABZ exhibited a markedly higher Higuchi constant (K_H_) than FBZ, indicating ~1.52-fold faster diffusion from the PCL core. Both systems displayed anomalous (non-Fickian) transport (0.43 < n < 0.85), revealing that release is governed by a combination of drug diffusion through the hydrophobic core and limited PCL chain relaxation/swelling. The higher n value for ABZ (0.824) indicates a stronger contribution from matrix relaxation, consistent with its lower lipophilicity (logP ≈ 3.0) and smaller micelle size (DLS 194 nm vs. 268 nm for FBZ). In contrast, the lower n for FBZ (0.532) reflects more diffusion-dominated release due to stronger hydrophobic interactions with the PCL core.

These kinetic differences directly correlate with the observed cytotoxicity profiles in MDA-MB-231 cells (Figure 9 and Figure 10). The rapid initial burst and higher cumulative release of ABZ (57% at 48 h) translated into greater long-term cytotoxicity of ABZ-loaded micelles at 48 h. Conversely, the slower, more sustained release of FBZ provided enhanced early delivery (24 h), overcoming its poor aqueous solubility and yielding better cytotoxicity at the shorter incubation time.

## 3. Materials and Methods

### 3.1. General Procedures

#### 3.1.1. Materials

Poly(ethylene glycol)-block-poly(ɛ-caprolactone) methyl ether (PEG-b-PCL), PCL average Mn~5000, PEG~5000, and albendazole were obtained from Sigma-Aldrich (St. Louis, MO, USA). Fenbendazole was supplied by Biovet AD (Peshtera, Bulgaria); 1,6-diphenyl-1,3,5-hexatriene (DPH) (Sigma-Aldrich), chloroform (Acros, Geel, Belgium), and tetrahydrofuran (THF) (Macron, Radnor, PA, USA) were used as received. 

#### 3.1.2. Methods

The hydrodynamic diameter was determined using dynamic light scattering with a Malvern Instruments Zetasizer Nano ZS (Malvern Panalytical, Malvern, UK). Measurements of the hydrodynamic diameter and particle size distribution were conducted at room temperature after filtering the aqueous micellar solution through a 0.45 μm filter. Ultraviolet–visible absorption spectra were recorded using a UV-Vis spectrophotometer (ONDA UV-31) (Giorgio Bormac S.r.l., Carpi, Italy). Transmission electron microscopy (TEM) analysis was carried out with a JEOL 2100 electron microscope (EOL Ltd., Tokyo, Japan) operating at an accelerating voltage of 200 kV and equipped with a digital camera. A drop of the micellar solution was placed on a copper grid coated with a carbon film, and the solvent was allowed to evaporate. Absorption spectra were also recorded with a Hewlett-Packard 8452A spectrophotometer (Hewlett-Packard, Waldbronn, Germany) in water.

### 3.2. Preparation of Polymeric Micelles and Drug-Loaded Micelles

Polymer micelles based on the PEG-b-PCL copolymer were prepared by adding a copolymer solution (20 mg) in THF (1 mL) to distilled water (19 mL) with stirring for 24 h at room temperature. The resulting solution was then dialyzed for 48 h using a Spectra/Por^®^ 7 MWCO 3500 dialysis membrane (Repligen Corp., Waltham, MA, USA), with frequent changes of deionized water. Micelles loaded with the drug were prepared following the same procedure by dissolving 2 mg of ABZ or FBZ and 20 mg of PEG-b-PCL copolymer in 1 mL of THF. The resulting solution was then added dropwise to 19 mL of distilled water while stirring for 24 h and subsequently dialyzed for 48 h using a dialysis membrane, with frequent changes of deionized water.

### 3.3. Drug Loading

A solution of ABZ or FBZ (2 mg) and PEG-b-PCL copolymer (20 mg) in THF (1 mL) was added dropwise to distilled water (19 mL) while stirring. The reaction mixture was stirred for 24 h at room temperature. Following this, the solution was dialyzed for 48 h using a dialysis membrane, with frequent changes of deionized water. The micellar solution was then filtered through a 0.45 μm filter, which was rinsed with THF. The amount of unencapsulated ABZ or FBZ in the THF rinsing fraction was determined using UV spectrophotometry at a wavelength of λ = 290 nm. The encapsulation efficiency (EE) was calculated from the following equation:

EE% = (Total mass of drug − Mass of free drug)/(Total mass of drug) × 100(3)

### 3.4. Drug Release

The release test was conducted using the dialysis method. A precise volume of micellar solution (2 mL) was placed inside a dialysis membrane, which was then immersed in buffered media at pH 7.4 and maintained at 37 °C. Samples were collected from the external medium, and the concentration of the released drug was measured spectrophotometrically at 290 nm to assess ABZ or FBZ release, using a pre-established calibration curve.

### 3.5. Cytotoxicity

#### 3.5.1. Cell Line and Culture Conditions

The human highly invasive triple-negative breast cancer (TNBC) cell line, MDA-MB-231, was obtained from the American Type Culture Collection (ATCC, Manassas, VA, USA). The cells were maintained in Dulbecco’s Modified Eagle Medium (DMEM) with high glucose (4.5 g/L), supplemented with 10% (*v*/*v*) foetal bovine serum (FBS), 2 mM L-glutamine, and 1% penicillin–streptomycin–amphotericin B (PSA) solution. Cell cultures were incubated in a humidified 5% CO_2_ atmosphere at 37 °C in 25 cm^2^ tissue culture flasks. The culture media were replenished every 48–72 h, and cells were passaged upon reaching 80% confluence using a standard trypsin–EDTA protocol to maintain logarithmic growth during the experiments.

#### 3.5.2. MTT Test for Cell Viability

The cytotoxic potential of PCL-PEG drug-loaded micelles was assessed using the colorimetric MTT (3-(4,5-dimethylthiazol-2-yl)-2,5-diphenyltetrazolium bromide) dye reduction assay, following the protocol described by Mosmann [41]. MDA-MB-231 cells were seeded in 96-well microplates at 5 × 10^3^ cells per well (100 µL per well) and allowed to adhere for 24 h. After attachment, cells were treated with cell culture medium containing a 10% (*v*/*v*) solution of PCL-PEG micelles loaded with drugs (ABZ or FBZ), yielding a final drug concentration of 10 µg/mL (0.01 mg/mL). Preliminary experiments were conducted with media containing 15% or 25% water solutions of PCL-PEG micelles loaded with drugs, but the higher water content, when used to replace cell media, itself resulted in substantial cytotoxicity, ranging from 30% (for 15% substitution) to more than 50% (for 25% substitution). Meanwhile, cells were treated with the drugs alone at the same concentration, 10 µg/mL. All samples were further incubated for 24 h and 48 h. As a control, untreated cells were used. After the incubation period, the cell medium was replaced with fresh medium (100 µL per well). Then, 20 µL of MTT solution (5 mg/mL in PBS) was added. Plates were further incubated for 3 h at 37 °C, and the formed formazan crystals were dissolved by adding 100 µL of solvent (5% formic acid in 2-propanol) per well and mixing. The absorbance was measured at 570 nm using a 96-well plate reader (Tecan Infinite F200 PRO, Tecan Austria GmbH, Salzburg, Austria). For each concentration, six replicates were performed in three independent experiments. A 5% formic acid solution in 2-propanol (100 mL) was used as the blank. Cell viability (%) was calculated as a percentage of the control value using the formula (OD treated/OD control) × 100%. Additional MTT experiments were conducted with drugs alone over specific concentration ranges for 24 h and 48 h to estimate IC50 values. The details are given in the Section 2.

Experimental groups were organized as follows:

Design of the experimental groups:

- Free ABZ/FBZ group (drug control group);
- Blank micelle group (carrier control group);
- Drug-loaded micelle group (experimental group);
- Non-treated cells (control group).

#### 3.5.3. Cell Morphology Assessment by Phase Contrast Microscopy

Cell morphology of treated MDA-MB-231 cells was examined using inverted phase-contrast microscopy (MEIJI, Chuo-ku, Japan) with the Optikam B1 Digital camera (Optica, Ponteranica, Italy).

#### 3.5.4. Data Analysis

The results are presented as mean values with standard deviation (SD) of the specified number of determinations. The statistical significance of differences was assessed using analysis of variance (ANOVA) with Tukey’s post hoc test, and results were considered statistically significant at *p* < 0.05. Analyses were conducted using GraphPad Prism software version 5 (GraphPad Software, Inc., La Jolla, CA, USA) and OriginPro version 2022 (OriginLab Corporation, Northampton, MA, USA).

## 4. Conclusions

The present study demonstrated that PEG-b-PCL micelles can successfully encapsulate the poorly water-soluble benzimidazole derivatives albendazole and fenbendazole with high encapsulation efficiency and nanosized dimensions. A comparative evaluation of ABZ- and FBZ-loaded micelles revealed differences in encapsulation behaviour, particle characteristics, drug release profiles, and in vitro anticancer activity. The obtained micellar systems exhibited distinct release behaviours, with FBZ showing slower release kinetics than ABZ, likely due to its higher lipophilicity and stronger interactions with the hydrophobic PCL core. In vitro studies against MDA-MB-231 triple-negative breast cancer cells demonstrated enhanced cytotoxic activity of the drug-loaded micelles compared to the free drugs, although different time-dependent effects were observed for ABZ- and FBZ-loaded micelles.

Further studies on cellular uptake, pharmacokinetics, biodistribution, and in vivo antitumour efficacy are required to better understand the therapeutic potential of these PEG-b-PCL nanosystems as a platform for delivering lipophilic anticancer agents.

## Figures and Tables

**Table 1:** Kinetic parameters of ABZ- and FBZ-loaded PEG-b-PCL micelles obtained from the Higuchi and Korsmeyer–Peppas models (initial burst phase, ≤60% release).

	Higuchi Model		Korsmeyer-Peppas Model			Release Mechanism
Drug	k_H_ [%/h^1/2^]	R^2^	Release Exponent (n)	Rate Constant (k)	R^2^	spherical micelles
ABZ-loaded micelles (0–6 h)	24.49	0.9437	0.824	0.1355	0.969	Anomalous (non-Fickian) transport
FBZ-loaded micelles (0–5 h)	16.14	0.9358	0.532	0.1675	0.894	Anomalous (non-Fickian) transport

## Data Availability

Data are available within the article.

## Abbreviations

The following abbreviations are used in this manuscript:

ABZ	Albendazole
FBZ	Fenbendazole
PEG-b-PCL	Poly(ethylene glycol)-block-poly(ε-caprolactone
